# PD-L1 expression is a promising predictor of survival in patients with advanced lung adenocarcinoma undergoing pemetrexed maintenance therapy

**DOI:** 10.1038/s41598-020-73013-3

**Published:** 2020-09-30

**Authors:** Yi Qin, Lili Jiang, Min Yu, Yanying Li, Xiaojuan Zhou, Yongsheng Wang, Youling Gong, Feng Peng, Jiang Zhu, Yongmei Liu, Yong Xu, Lin Zhou, You Lu, Meijuan Huang

**Affiliations:** 1 Department of Thoracic Oncology, Cancer Center, West China Hospital, Sichuan University, Chengdu, 610041 China; 2 Department of Pathology, West China Hospital, Sichuan University, Chengdu, 610041 China

**Keywords:** Non-small-cell lung cancer, Non-small-cell lung cancer, Cancer therapy, Tumour biomarkers

## Abstract

This study aimed to identify potential predictive factors for the survival of advanced lung adenocarcinoma patients undergoing pemetrexed maintenance therapy. 122 advanced lung adenocarcinoma patients who received pemetrexed maintenance therapy were retrospectively analyzed. Kaplan–Meier method with Log-rank test was used for survival analysis. Univariate and multivariate Cox regression were performed to evaluate prognostic factors for overall survival (OS) and progression-free survival (PFS). Bivariate correlation analysis was used for exploratory purpose. For the whole cohort of 122 patients, median PFS was 11.97 months (95% CI 10.611–13.329) and estimated median OS was 45.07 months (95% CI 31.690–58.450). The mPFS of ALK-positive patients was superior to negative patients (18.27 vs. 11.90 months; *P*  = 0.039). Patients with ECOG PS 0 (14.4 vs. 11.1 months; p = 0.040) and patients with single-organ metastasis (19.0 vs. 11.0 months; p = 0.014) had prolonged median PFS. Compared with the low PD-L1 expression group, PFS of high PD-L1 expression group were improved (13.6 vs. 11.1 months, p = 0.104, at 1% cut-off; 17.5 vs. 11.1 months, p = 0.009, at 10% cut-off; and 27.5 vs. 11.4 months, p = 0.005, at 50% cut-off). No differences were found between EGFR positive and negative patients. PD-L1 expression was an independent prognostic factor for both PFS and OS times (PFS: HR, 0.175; *P*  = 0.001; OS: HR, 0.107; *P*  = 0.036). Bivariate correlation showed a significant positive correlation between PD-L1 expression and PFS (correlation coefficient R = 0.485, *P*  < 0.001). High PD-L1 expression could be a potential effective predictor for favorable survival of advanced lung adenocarcinoma patients undergoing pemetrexed maintenance therapy.

## Introduction

Although advances in targeted therapy and immunotherapy are revolutionizing the landscape of cancer treatment, chemotherapy remains an integral element of the therapeutic armamentarium for most patients with advanced lung cancer^1^. Now increasing evidence shows that chemotherapy can exert anti-tumor effects through modulating tumor immunity, indicting its potential for coordination with other therapies^2–5^. Thus, the combinations of chemotherapy with anti-angiogenic therapy, targeted therapy and immunotherapy have been extensively evaluated to further improve outcomes of patients with advanced non-small-cell lung cancer (NSCLC)^6–9^.

Pemetrexed is a multi-targeted antifolate cytotoxic agent disrupting folate-dependent metabolic processes in nucleotide and DNA synthesis^10,11^. Due to its better tolerability and lower toxicity compared with other cytotoxic drugs, pemetrexed has validated evidence of maintenance therapy and is more attractive in combination therapies in advanced non-squamous NSCLC^12,13^. Meanwhile, pemetrexed shows synergistic effect with programmed death-1 (PD-1)/programmed death ligand 1 (PD-L1) axis inhibitors^8,14^. Of note, pemetrexed plus carboplatin and anti-PD-1 (pembrolizumab) regimen in NSCLC is the first approved chemotherapy and immunotherapy combination. However, the useful biomarker for the efficacy of pemetrexed-based treatment remains unknown^15^.

Here, we evaluated all patients receiving pemetrexed maintenance therapy in our center, focusing on the association between clinical outcomes and various factors, especially PD-L1 expression, for additional insights into the association will help to advance the design of combination strategies.

## Methods

### Patients

Eligible patients for this study had histological or cytological confirmation of lung adenocarcinoma, stage IV according to the tumor–nodes–metastasis (TNM) criteria (7th edition, AJCC criteria 2009); underwent at least four cycles of induction pemetrexed-based chemotherapy and received pemetrexed maintenance therapy at the Department of Thoracic Oncology of West China Hospital, Sichuan University. From January 2011 to June 2017, a total of 122 patients from our institution fulfilling all those criteria were enrolled. We collected data on age, sex, Eastern Cooperative Oncology Group (ECOG) performance status (PS), smoking history, treatment schedule, treatment response, epidermal growth factor receptor (EGFR) status, anaplastic lymphoma kinase (ALK) status, PD-L1 expression, progression-free survival (PFS), and overall survival (OS). The therapeutic response was assessed using the Response Evaluation Criteria in Solid Tumors (RECIST) version 1.1. PFS was calculated from the initiation of pemetrexed chemotherapy to disease progression or death. OS was defined as the period from the initiation of pemetrexed chemotherapy to death. Patients were followed-up until April 2019. The primary endpoint was PFS and the secondary endpoint was OS. This retrospective study was approved by the ethics committee of West China Hospital Sichuan University (No. 523), and written informed consent requirement was waived.

### Statistical methods

Kaplan–Meier method and the log-rank test were used to conduct survival analysis. Predictors for survival were tested by univariate and multivariate analyses using the Cox proportional hazard regression model with backward elimination. Bivariate correlation analysis was used to evaluate the correlation between two variables. A two-tailed *p-*value of less than 0.05 was considered statistically significant. Analyses were performed using SPSS 22.0 software (IBM Corp., Armonk, NY).

### Ethical approval

This study was approved by the Biomedical Ethics Committee of West China Hospital of Sichuan University. A copy of the approval document is available for review by the Editorial team. All applicable international, national, and institutional guidelines for the care and use of animals were followed. All procedures performed in studies involving human participants were in accordance with the ethical standards of the institutional and national research committee and with the 1964 Helsinki declaration and its later amendments or comparable ethical standards.

## Results

### Patient characteristics

In this cohort of 122 patients, the median age was 58 years, 62 (50.8%) were male and 73 (59.8%) were never smokers. Most patients had an ECOG PS of 0–1 (98.4%). 36 patients harbored EGFR mutations and 10 patients harbored ALK translocations. All EGFR-positive patients had received EGFR tyrosine kinase inhibitors (TKIs) in the course of the disease, before or after pemetrexed treatment. All ALK-positive patients but two, who continued to benefit from first-line pemetrexed maintenance chemotherapy, received ALK TKIs in the course of the disease. 81 patients examined PD-L1 immunohistochemistry, and 8 of them had high PD-L1 expression (≥ 50%). The clinicopathological characteristics of the enrolled patients are shown in Table 1.

**Table 1:** Baseline demographics and disease characteristics.

Characteristics	No. of patients (%)
**Gender**	
Male	62 (50.8)
Female	60 (49.2)
**Age (years)**	
Median	58
Range	31–79
< 65 years	89 (73.0)
≥ 65 years	33 (27.0)
**Smoking history**	
Former or current smoker	49 (40.2)
Never smoker	73 (59.8)
**ECOG PS**	
0	64 (52.5)
1	56 (45.9)
2	2 (1.6)
**Tumor size**	
T1/T2	48 (39.3)
T3/T4	74 (60.7)
**Lymph node**	
N0/N1	29 (23.8)
N2/N3	93 (76.2)
**Metastasis**	
Single organ	34 (27.9)
Multiple organs	88 (72.1)
**EGFR status**	
Negative	77 (63.1)
Positive	36 (29.5)
Unknown	9 (7.4)
**ALK status**	
Negative	83 (68.0)
Positive	10 (8.2)
Unknown	29 (23.8)
**PD-L1 expression**	
< 1%	51 (41.8)
≥ 1%, < 10%	8 (6.6)
≥ 10%, < 50%	14 (11.5)
≥ 50%	8 (6.6)
Unknown	41 (33.6)
**Therapy line of pemetrexed**	
First	77 (63.1)
Second	36 (29.5)
Third/fourth	9 (7.4)
**Chest radiotherapy**	
Yes	25 (20.5)
No	97 (79.5)
**Targeted therapy for the EGFR-positive**	
Before pemetrexed	27 (75)
After pemetrexed	9 (25)
**Targeted therapy for the ALK-positive**	
Before pemetrexed	2 (20)
After pemetrexed	6 (60)
Never	2 (20)

ECOG, Eastern Cooperative Oncology Group; PS, performance status; EGFR, epidermal growth factor receptor; ALK, anaplastic lymphoma kinase; PD-L1, programmed death ligand 1.

### Treatment

All patients received 4–6 cycles of pemetrexed-based induction chemotherapy and 77 (63.1%) of them were on the first line. 25 patients (20.5%) received chest radiotherapy before or during pemetrexed treatment. Regimens in the induction phase were pemetrexed/cisplatin in 78 patients (64%); pemetrexed/carboplatin in 30 patients (25%); pemetrexed/oxaliplatin in 3 patients (2%) and pemetrexed in 11 patients (9%). 122 patients received pemetrexed maintenance therapy with a median of 4 (range = 1–63 cycles) cycles.

### Survival time

**Figure 1:**
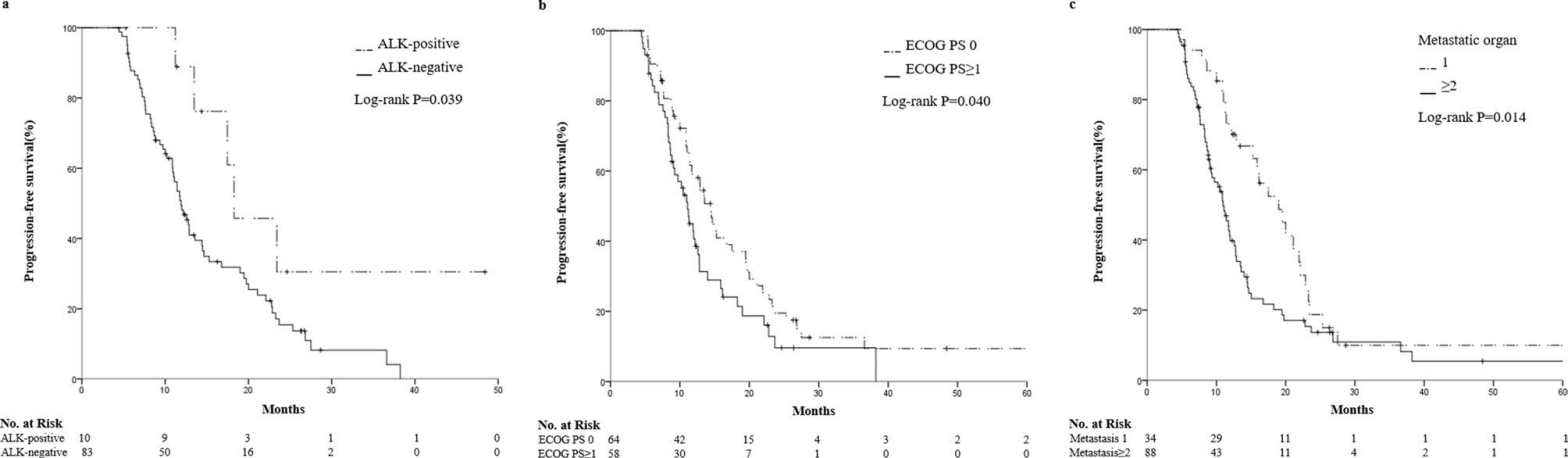
Kaplan–Meier curves of PFS in advanced lung adenocarcinoma patients undergoing pemetrexed maintenance therapy according to ALK status (**a**), ECOG PS (**b**), and metastatic organ (**c**). PFS, progression-free survival; ALK, anaplastic lymphoma kinase; ECOG, Eastern Cooperative Oncology Group; PS, performance status.

**Figure 2:**
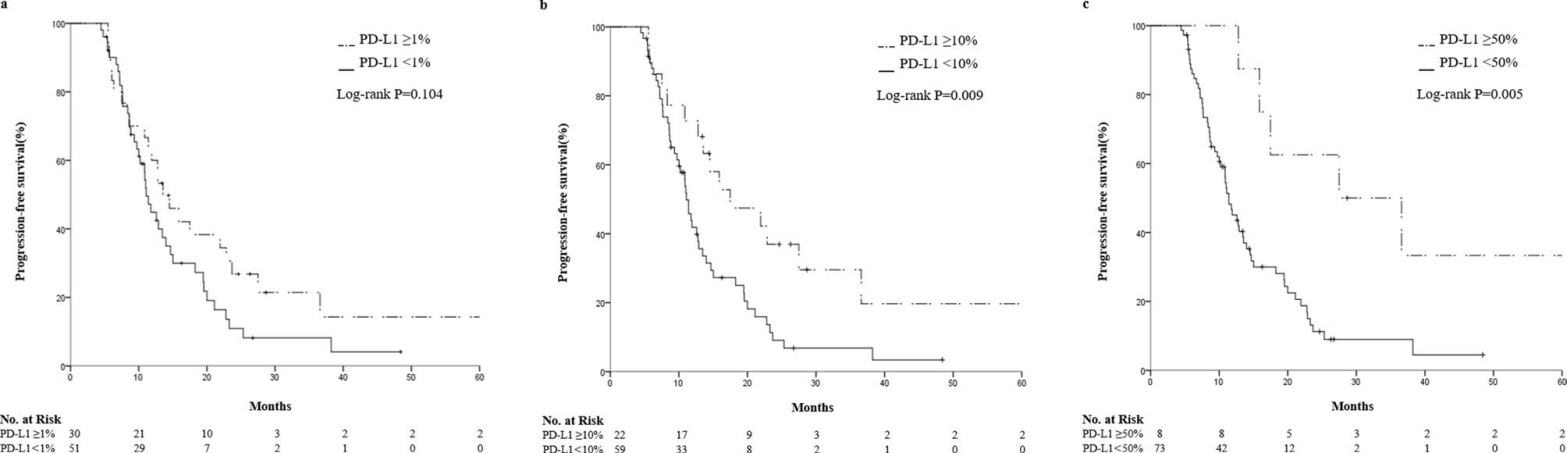
Kaplan–Meier curves of PFS in advanced lung adenocarcinoma patients undergoing pemetrexed maintenance therapy according to PD-L1 expression. PFS time for the (**a**) 1%, (**b**) 10% and (**c**) 50% cut-off. PFS, progression-free survival; PD-L1, programmed death ligand 1.

After a median follow-up of 30 months, 94 patients (77.0%) and 41 patients (33.6%) reached the end point of disease progression and death, respectively. Median PFS was 11.97 months (95% CI 10.611–13.329) and estimated median OS was 45.07 months (95% CI 31.690–58.450) for the whole cohort. The median PFS of ALK-positive patients was superior to negative patients (18.3 vs. 11.9 months; p = 0.039; Fig. 1a). Patients with ECOG PS 0 (14.4 vs. 11.1 months; p = 0.040; Fig. 1b) and patients with single-organ metastasis (19.0 vs. 11.0 months; p = 0.014; Fig. 1c) had prolonged median PFS. Compared with the low PD-L1 expression group, at 1, 10 and 50% cut-off values, the PFS of high PD-L1 expression group were improved (13.6 vs. 11.1 months, p = 0.104, at 1% cut-off; 17.5 vs. 11.1 months, p = 0.009, at 10% cut-off; and 27.5 vs. 11.4 months, p = 0.005, at 50% cut-off; Fig. 2). EGFR mutations did not show significant correlation with PFS (11.0 vs. 12.8 months; p = 0.097). The Kaplan Meier method is immature for OS analysis.

### Predictive factors for PFS and OS

We performed univariate and multivariate analyses using Cox proportional hazards regression model to evaluate the potential risk factors for PFS and OS (Table 2, Supplementary Table S1). Univariate survival analysis revealed that ECOG PS 0, single-organ metastasis, PD-L1 ≥ 50% were significantly associated with longer PFS times (P = 0.042, 0.015 and 0.005, respectively). ECOG PS 0 was also associated with longer OS times (P = 0.009).

**Table 2:** Univariate and multivariate analysis of prognosis factors associated with survival.

Parameters	PFS		OS	
	HR (95% CI)	P-value	HR (95% CI)	P-value
**Univariate analysis**				
Age (≥ 65 vs. < 65)	0.724 (0.449–1.168)	0.186	0.842 (0.412–1.720)	0.636
Gender (male vs. female)	1.011 (0.673–1.521)	0.956	0.876 (0.471–1.626)	0.876
Smoking (yes vs. no)	0.947 (0.624–1.438)	0.800	0.837 (0.441–1.586)	0.585
ECOG PS (0 vs. ≥ 1)	**0.653 (0.432–0.985)**	**0.042**	**0.423 (0.221–0.808)**	**0.009**
Tumor size (T1-2 vs. T3-4)	0.879 (0.579–1.334)	0.545	0.777 (0.407–1.485)	0.445
Lymph node (N0-1 vs. N2-3)	1.100 (0.680–1.780)	0.698	1.019 (0.485–2.143)	0.959
Metastatic organ (1 vs. ≥ 2)	**0.568 (0.360–0.897)**	**0.015**	0.656 (0.321–1.340)	0.247
Therapy line (1–2 vs. 3–4)	0.787 (0.364–1.705)	0.544	0.950 (0.292–3.088)	0.932
Radiotherapy (yes vs. no)	0.846 (0.523–1.368)	0.495	0.675 (0.317–1.438)	0.309
EGFR (positive vs. negative)	1.440 (0.934–2.219)	0.099	0.806 (0.392–1.657)	0.557
ALK (positive vs. negative)	0.409 (0.165–1.017)	0.054	0.752 (0.225–2.517)	0.644
PD-L1 (≥ 50% vs. < 50%)	**0.261 (0.102–0.670)**	**0.005**	0.215 (0.028–1.630)	0.137
**Multivariate analysis**				
Age (≥ 65 vs. < 65)	**0.560 (0.340–0.924)**	**0.023**	N/A	N/A
ECOG PS (0 vs. ≥ 1)	N/A	N/A	**0.459 (0.236–0.894)**	**0.022**
Metastatic organ (1 vs. ≥ 2)	**0.586 (0.368–0.934)**	**0.025**	N/A	N/A
ALK (positive vs. negative)	**0.290 (0.114–0.738)**	**0.009**	N/A	N/A
PD-L1 (≥ 50% vs. < 50%)	**0.175 (0.062–0.489)**	**0.001**	**0.107 (0.013–0.868)**	**0.036**

Bold values indicate P-value < 0.05.

PFS, progression-free survival; OS, overall survival; HR, hazard ratio; CI, confidence interval; ECOG, Eastern Cooperative Oncology Group; PS, performance status; EGFR, epidermal growth factor receptor; ALK, anaplastic lymphoma kinase; PD-L1, programmed death ligand 1; N/A, not applicable.

Multivariate analysis demonstrated that age (≥ 65 vs. < 65, HR: 0.560, 95% CI 0.340–0.924, P = 0.023), metastatic organ (1 vs. ≥ 2, HR: 0.586, 95% CI 0.368–0.934, P = 0.025), ALK status (positive vs. negative, HR: 0.290, 95% CI 0.114–0.738, P = 0.009) and PD-L1 expression (≥ 50% vs. < 50%, HR: 0.175, 95% CI 0.062–0.489, P = 0.001) were independent risk factors for PFS. ECOG PS (0 vs. ≥ 1, HR: 0.459, 95% CI 0.236–0.894, P = 0.022) and PD-L1 expression (≥ 50% vs. < 50%, HR: 0.107, 95% CI 0.013–0.868, P = 0.036) were significant predictive factors for OS.

**Figure 3:**
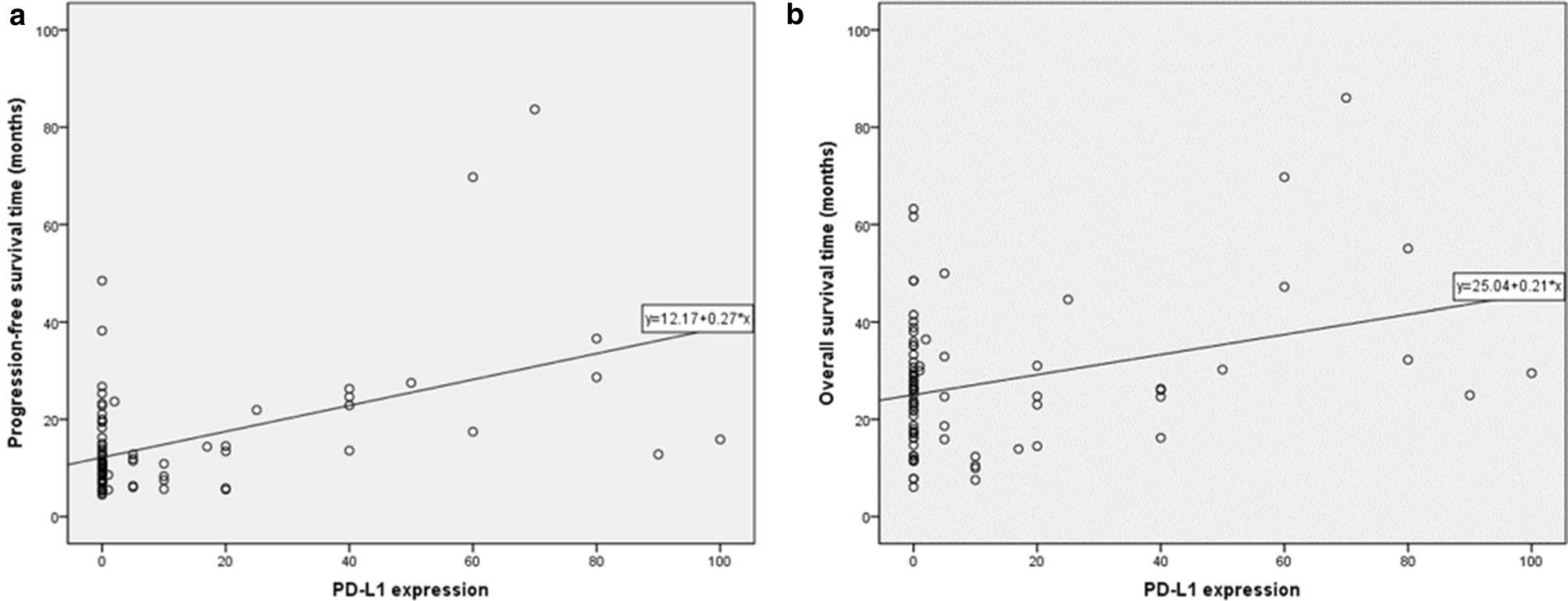
Scatter plots of the correlation between PD-L1 expression and PFS (**a**) and between PD-L1 expression and OS (**b**). PFS, progression-free survival; OS, overall survival; PD-L1, programmed death ligand 1.

To further explore the association between PD-L1 expression and survival in advanced lung adenocarcinoma patients treated with pemetrexed, a bivariate correlation analysis was performed. It showed that PFS and OS were positively correlated with PD-L1 expression (correlation coefficient R = 0.485, P < 0.001, for PFS; R = 0.330, P = 0.003, for OS). The scatter plots of PD-L1 expression and survival time were shown in Fig. 3. These results indicated that high PD-L1 expression may predict prolonged survival of pemetrexed-based chemotherapy.

## Discussion

PD-L1, as an immunoinhibitory molecule that inhibits T cell-mediated immune responses, is mainly expressed on the surface of tumor cells and antigen-presenting cells in various solid malignancies including lung cancer^16,17^. However, the prognostic value of PD-L1 expression in NSCLC is controversial. Its positive and negative prediction values are reported^18–23^. As for certain chemotherapy regimen, the role of PD-L1 in prognosis is rarely evaluated.

Our results suggested high PD-L1 expression was an independent protective predictor of prognosis for pemetrexed maintenance treatment. Consistent conclusion has been reported in another retrospective study: a total of 56 patients with advanced lung adenocarcinoma, who received first-line pemetrexed-based chemotherapy, the median PFS of PD-L1 positive patients (5% cut-off) was significantly longer compared to the negative (6.4 vs. 3.9 months; P = 0.008), although whether the patients received pemetrexed maintenance therapy has not been described^24^. As the pemetrexed maintenance therapy significantly prolongs the survival, we supposed that the gaps between the advantage group and disadvantage group would be more obvious in the maintenance cohort, which might contribute to recognize predictors of prognosis, and that was why we enrolled patients who received maintenance therapy. Furthermore, research shows that cisplatin upregulated PD-L1 expression by tumor cells in NSCLC^25,26^. Together with our findings, this phenomenon could favor the results of consecutive pemetrexed treatment. Indeed, notable gaps between PD-L1 high and low were observed in our study (27.5 vs. 11.4 months, p = 0.005, at 50% cut-off).

Moreover, the relationship between PD-L1 and chemotherapy efficacy is not only found in pemetrexed regimen. Similarly, a recent study shows that PD-L1 polymorphisms can predict clinical outcomes of first-line paclitaxel-cisplatin chemotherapy in NSCLC^27^. Now the role of biomarker of PD-L1 is being enriched^28^ and the complex effect of PD-L1 signaling is consistent with its imperfect correlation with efficacy of immune checkpoint inhibitors^29^. Thus, due to the complexity, it is critical to reassess and gain insight into the value of PD-L1, especially in combination therapies.

Finally, it is noteworthy that pemetrexed has immunomodulatory effect. It is reported that pemetrexed enhances intratumor immune responses through tumor intrinsic mechanisms including immunogenic cell death, T-cell-intrinsic mechanisms enhancing mitochondrial biogenesis leading to increased T-cell infiltration/activation along with modulation of innate immune pathways^30^. Although the inherent mechanisms remain unclear, these results show a multidirectional relationship between pemetrexed and immunity, which is not only the basis of the combination of pemetrexed and immunotherapy, but also the direction worth studying in the future.

In conclusion, our results indicated that high PD-L1 expression could be a potential effective predictor for the favorable outcome of advanced lung adenocarcinoma patients sustaining pemetrexed treatment. More relevant studies in a larger cohort are warranted to validate the complex relationship between pemetrexed and PD-1/PD-L1 pathway and explore the potential mechanisms.

## Supplementary information

Supplementary Information.
