# Supplementary material for: PD-L1 expression is a promising predictor of survival in patients with advanced lung adenocarcinoma undergoing pemetrexed maintenance therapy
Source: Sci Rep. 2020 Sep 30;10:16150. doi: 10.1038/s41598-020-73013-3 (PMC7527332; doi:10.1038/s41598-020-73013-3)
Supplement: Supplementary file 1 — Supplementary Information. [file 41598_2020_73013_MOESM1_ESM.docx]

**Supplementary Information**

**PD-L1 expression is a promising predictor of survival in patients with advanced lung adenocarcinoma undergoing pemetrexed maintenance therapy**

Yi Qin^1#^, Lili Jiang^2#^, Min Yu^1^, Yanying Li^1^, Xiaojuan Zhou^1^, Yongsheng Wang^1^, Youling Gong^1^, Feng Peng^1^, Jiang Zhu^1^, Yongmei Liu^1^, Yong Xu^1^, Lin Zhou^1^, You Lu^1^, Meijuan Huang^1^*

#These authors contributed equally.

**Authors’ Affiliation**

1. Department of Thoracic Oncology, Cancer Center, West China Hospital, Sichuan University, Chengdu, 610041, China;
2. Department of Pathology, West China Hospital, Sichuan University, Chengdu, 610041, China;

***Corresponding author:**

**Meijuan Huang**

Professor of Department of Thoracic Oncology

West China Hospital, Cancer Center

Sichuan University

Chengdu, China 610041

Email: [Huang_MJ@outlook.com](mailto:Huang_MJ@outlook.com)

Supplementary **Table S1.** Univariate and multivariate analysis of survival-related prognosis factors at PD-L1 cut-off values of 10% and 1%**.**

| **Parameters** | **PFS** | | |  | **OS** | | |
| --- | --- | --- | --- | --- | --- | --- | --- |
|  | **HR (95% CI)** | **P-value** | **HR (95% CI)** | | | **P-value** |  |
| **Univariate analysis** |  |  |  | | |  |  |
| PD-L1 (≥10% vs <10%) | **0.445 (0.247-0.802)** | **0.007** | **0.120 (0.016-0.896)** | | | **0.039** |  |
| PD-L1 (≥1% vs <1%) | 0.631 (0.376-1.059) | 0.081 | **0.240 (0.070-0.817)** | | | **0.022** |  |
|  |  |  |  | | |  |  |
| **Multivariate analysis (1)** |  |  |  | | |  |  |
| Age (≥65 vs <65) | 0.648 (0.398-1.054) | 0.081 | N/A | | | N/A |  |
| ECOG PS (0 vs ≥1) | N/A | N/A | **0.491 (0.253-0.956)** | | | **0.036** |  |
| Metastatic organ (1 vs ≥2) | **0.601 (0.377-0.956)** | **0.032** | N/A | | | N/A |  |
| ALK (positive vs negative) | **0.362 (0.144-0.908)** | **0.030** | N/A | | | N/A |  |
| PD-L1 (≥10% vs <10%) | **0.446 (0.239-0.832)** | **0.011** | **0.081 (0.010-0.630)** | | | **0.016** |  |
|  |  |  |  | | |  |  |
| **Multivariate analysis (2)** |  |  |  | | |  |  |
| Age (≥65 vs <65) | N/A | N/A | N/A | | | N/A |  |
| ECOG PS (0 vs ≥1) | N/A | N/A | **0.422 (0.216-0.828)** | | | **0.012** |  |
| Metastatic organ (1 vs ≥2) | **0.529 (0.334-0.838)** | **0.007** | N/A | | | N/A |  |
| ALK (positive vs negative) | **0.374 (0.150-0.931)** | **0.035** | N/A | | | N/A |  |
| PD-L1 (≥1% vs <1%) | N/A | N/A | **0.167 (0.047-0.589)** | | | **0.005** |  |

PFS, progression-free survival; OS, overall survival; HR, hazard ratio; CI, confidence interval; ECOG, Eastern Cooperative Oncology Group; PS, performance status; EGFR, epidermal growth factor receptor; ALK, anaplastic lymphoma kinase; PD-L1, programmed death ligand 1; N/A, not applicable.
